# Topology dependence of skyrmion Seebeck and skyrmion Nernst effect

**DOI:** 10.1038/s41598-022-10550-z

**Published:** 2022-04-26

**Authors:** Markus Weißenhofer, Ulrich Nowak

**Affiliations:** Department of Physics, University of Konstanz, 78457 Konstanz, Germany

**Keywords:** Nonlinear phenomena, Magnetic properties and materials, Surfaces, interfaces and thin films, Atomistic models

## Abstract

We explore the dynamics of skyrmions with various topological charges induced by a temperature gradient in an ultra-thin insulating magnetic film. Combining atomistic spin simulations and analytical calculations we find a topology-dependent skyrmion Seebeck effect: while skyrmions and antiskyrmions move to the hot regime, a topologically trivial localized spin structure moves to the cold regime. We further reveal the emergence of a skyrmion Nernst effect, i.e. finite, topology-dependent velocities transverse to the direction of the temperature gradient. These findings are in agreement with accompanying simulations of skyrmionic motion induced by monochromatic magnon currents, allowing us to demonstrate that the magnonic spin Seebeck effect is responsible for both, skyrmion Seebeck and Nernst effect. Furthermore we employ scattering theory together with Thiele’s equation to identify linear momentum transfer from the magnons to the skyrmion as the dominant contribution and to demonstrate that the direction of motion depends on the topological magnon Hall effect and the topological charge of the skyrmion.

## Introduction

Magnetic skyrmions are localized spin structures embedded in a homogeneous magnetic phase with nontrivial topology^1^ that can be characterized by a topological charge, $Q= -1/4\pi \int \mathrm {d}^2 r\ \mathbf {S} \cdot (\partial _x \mathbf {S} \times \partial _y \mathbf {S})$, where $\mathbf {S}$ is a smooth unit vector field pointing in the direction of the magnetic moment. Since the first detection of a skyrmion lattice in MnSi^2^, skyrmions have been found experimentally in various other bulk systems^3–8^ and in thin films^9–11^. The most widely considered mechanism for the stabilization of skyrmions is based on the Dzyaloshinsky-Moriya interaction (DMI)^12,13^ which is present in systems with broken inversion symmetry^1^. Competing with isotropic Heisenberg exchange, uniaxial anisotropy and Zeeman terms, which all favor collinear alignment of the spins, this chiral interaction can give rise to metastable isolated skyrmionic spin structures. Another mechanism that stabilizes skyrmions involves frustration of the Heisenberg exchange, i.e. the competition between ferro- and antiferromagnetic interactions for different neighbors in the crystal, which leads to the formation of modulated spin structures^14–19^. Theoretical works^20,21^ demonstrated that the interplay between frustrated Heisenberg exchange and DMI can lead to the formation of metastable isolated skyrmionic spin structures with various topological charges ranging from $-3$ to 3 in (Pt_0.95_Ir_0.05_)/Fe/Pd(111), which is the system investigated in this study. In addition, a pronounced impact of topology on the current-driven^22^ as well as Brownian dynamics^23^ of these skyrmionic spin structures was revealed. In the following, we will focus on the dynamics of these skyrmionic spin structures induced by a temperature gradient.

The dynamics of magnetic solitons induced by temperature gradients were first examined for domain walls. Both theoretical^24–26^ and experimental studies^27,28^ have revealed that ferromagnetic domain walls move towards the hot end of the system, due to a combination of magnonic and entropic effects. In contrast, a recent experimental study found that skyrmions in a conducting ferromagnet propagate from the hot to the cold regime, caused by the interplay of entropic forces, magnonic spin torques and thermal spin-orbit torques^29^. This is different in insulating systems, where thermal spin-orbit torques are missing and skyrmions were found to move towards the hot regime, along with a small transverse velocity, both theoretically^30,31^ and experimentally^32^. The latter result was explained phenomenologically via the magnonic spin Seebeck effect^33^, which causes a thermal magnon current traveling from the hot to the cold regime and exerting an effective force on the skyrmion^34^.

Here, we focus on insulating systems, neglecting the possible contributions of thermal electric currents. We investigate the directional motion of skyrmionic spin structures with various topological charges induced by a temperature gradient by means of atomistic spin simulations based on the stochastic Landau-Lifshitz-Gilbert equation^35^. We reveal that skyrmionic spin structures move either towards the hot or the cold end, depending on their topological charge, and reveal that the motion towards the hot end is of topological origin. We further demonstrate the emergence of a finite, topology-dependent velocitiy transverse to the direction of the temperature gradient. We summarize the observed longitudinal dynamics under the term *skyrmion Seebeck effect* and the transverse motion under the term *skyrmion Nernst effect*, in analogy to the classical Seebeck and Nernst effect^36,37^. Our findings for the dynamics of skyrmions in temperature gradients are well explained by accompanying simulations of the scattering of a monochromatic magnon current at the skyrmionic spin structure. Using an analytical approach based on Thiele’s equation^38^ and scattering theory we identify linear momentum transfer as the dominant mechanism for the skyrmion Seebeck and Nernst effect.

## Results

### Skyrmion Seebeck and skyrmion Nernst effect

**Figure 1:**
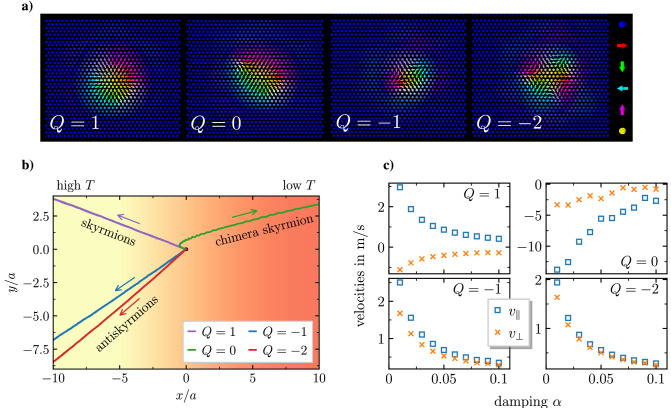
Topology dependence of the skyrmion Seebeck and skyrmion Nernst effect. (**a**) Equilibrium spin configurations of skyrmionic spin structures in (Pt$_{0.95}$/Ir$_{0.05}$)/Fe/Pd(111) with topological charges as labeled. The colors indicate the orientation of the spin vectors. (**b**) Averaged trajectories for the dynamics of skyrmionic spin structures with different topological charges in a temperature gradient $\mathbf {\nabla } T \parallel -\mathbf {e}_x$ with $\alpha =0.01$. Arrows indicate the direction of motion. (**c**) Velocities parallel and perpendicular to the direction of the temperature gradient for skyrmionic spin structures with different topological charges.

We simulate a spin model for a (Pt_0.95_Ir_0.05_)/Fe bilayer on a Pd(111) surface, a model which can stabilize skyrmionic structures with different topological charges, focusing on skyrmions ($Q=1$), antiskyrmions ($Q=-1$), second order antiskyrmions ($Q=-2$) and the topologically trivial chimera skyrmions ($Q=0$), see Fig. 1a. Note that the topological charges of the spin configurations in the spin model are evaluated using the discrete version of the formula for *Q*, see for example Ref.^39^. Skyrmions with $Q=\pm 3$ and $Q=2$ that have also been observed in this system^21^ were omitted in this study due to their limited thermal stability. Since it is topologically trivial, the $Q=0$ structure depicted in Fig. 1a is not actually a skyrmion. As it consists of one half of a skyrmion and one half of an antiskyrmion, it has been, however, referred to as chimera skyrmion^20^, inspired by greek mythology. Nonetheless, we use the term *skyrmionic spin structures* to include all four types of structures investigated here. We also want to emphasize that the DMI present in the system under consideration, (Pt_0.95_Ir_0.05_)/Fe/Pd(111), breaks the symmetry between skyrmions with positive and negative topological charges, which is responsible for the drastic difference in stability between skyrmions with $Q=\pm 2$, see also Ref.^20^, and which gives rise to the difference in shape and size, and consequently in the dynamics, between skyrmions with $Q=\pm 1$. Furthermore we want to note that a possible impact of the orientation of the skyrmions induced by an anisotropic shape, as reported for example in Ref.^40^, is neglected here, as their orientation is unchanged during their motion.

Figure 1b shows the averaged trajectories of skyrmionic spin structures driven by a temperature gradient $\mathbf {\nabla } T \parallel -\mathbf {e}_x$. It is the key finding of this study. All skyrmionic spin structures start at (0, 0) and, as time progresses, they drift away from their initial position. We find that, depending on the topological charge, they move in completely different directions. The longitudinal motion, which will be referred to as *skyrmion Seebeck effect* in analogy to the classical Seebeck effect^36^, depends on topology: while skyrmionic spin structures with finite topological charge are driven towards the high temperature region, the topologically trivial chimera skyrmion drifts towards the low temperature region. Furthermore, we find that the velocity transverse to the direction of the temperature gradient is nonzero for all types of skyrmions and that its sign depends on the topological charge: while antiskyrmions move towards the bottom in Fig. 1b, the skyrmion and the chimera skyrmion move towards the top. The occurrence of transverse velocities is quite similar to the skyrmion Hall effect where the skyrmionic spin structures are driven by a spin-polarized electrical current^22,41^. Since here the transverse velocities are induced by a temperature gradient, we term this phenomenon *skyrmion Nernst effect*, in analogy to the classical and spin Nernst effect^37,42^.

**Figure 2:**
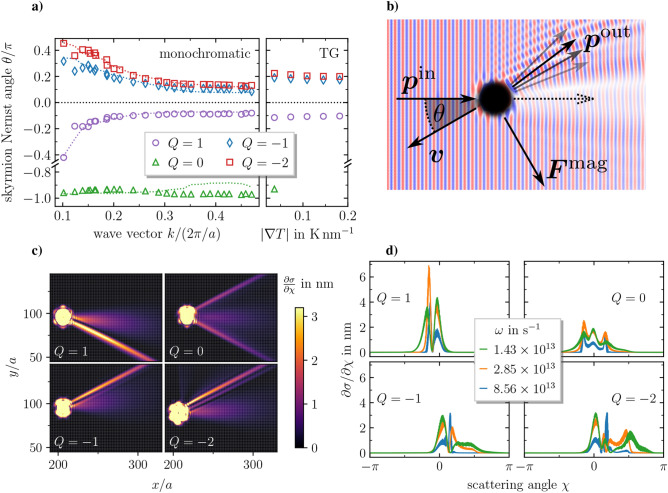
Skyrmionic spin structures driven by monochromatic magnon currents. (**a**) Skyrmion Nernst angle $\theta$ versus absolute value of the wave vector $\mathbf {k}$ of the monochromatic magnon current and strength of the temperature gradient $|\mathbf {\nabla } T|$ for skyrmionic spin structures with different topological charges at $\alpha =0.01$. Open symbols correspond to the simulated values and dotted lines are the predictions from Eq. (3) based on the differential cross sections. Note, that due to the lack of topological protection the chimera skyrmion gets destroyed more easily by a finite temperature as compared to the other skyrmions and it was found to be stable only at the lowest value for the temperature gradient that we simulated. (**b**) Illustration of magnons scattering at an antiskyrmion. The magnons are emitted from the left with momentum $\mathbf {p}^\mathrm {in}$ and, after being scattered, leave the system with $\mathbf {p}^\mathrm {out}$, giving rise to the force $\mathbf {F}^\mathrm {mag}$ via Eq. (1). For finite *Q* and small $\alpha$, Thiele’s Eq. (2) predicts that the velocity $\mathbf {v}$ is perpendicular to the force. For the antiskyrmion, the direction of the velocity can hence be obtained by a clockwise rotation of $\mathbf {F}^\mathrm {mag}$. (**c**) Differential cross sections as a function of spatial coordinates *x*, *y* obtained from simulation data for $\omega ={8.56\times 10^{13}}{\mathrm{s}^{-1}}$ and $\alpha =0.01$. The color coding has a cutoff value of $\partial \sigma /\partial \chi ={3.2}{\mathrm{nm}}$ for the sake of visibility, because our method of calculating the differential cross section (see Supplemental Material) yields very large values in the vicinity of the skyrmion core, where it is not applicable anyway. (**d**) Differential cross sections as a function of the scattering angle $\chi$ obtained from simulation data for $\alpha =0.01$. They are obtained by selecting all values of $\partial \sigma /\partial \chi$ within an annulus of width 10*a* with an outer diameter of 40*a*.

The surprising observation that skyrmionic spin structures move in different directions depending on their topological charge was found to be independent of the Gilbert damping $\alpha$. The velocities parallel and perpendicular to the direction of the temperature gradient, $v_\parallel = \mathbf {v} \cdot (-\mathbf {e}_x)$ and $v_\perp = \mathbf {v} \cdot (-\mathbf {e}_y)$, for all skyrmionic spin structures under investigation are displayed versus $\alpha$ in Fig. 1c, revealing an inverse proportionality for all velocities. This is in contrast to an earlier work on this topic^30^, where an inverse proportionality was reported solely for the longitudinal velocity. We further find that the velocities of the chimera skyrmion are much larger as compared to skyrmions and antiskyrmions. Together with the fact that chimera skyrmions are more easily deformed, which gives rise to inertial effects, these peculiar dynamics are responsible for its initially nonlinear trajectory depicted in Fig. 1b, in contrast to the other skyrmionic structures. Note that the chimera skyrmion only reaches a regime of rigid body motion with constant velocity after having left the section shown in Fig. 1b and, as a consequence, its skyrmion Nernst angle is smaller than it appears in this figure , cf. Fig. 2a.

### Magnon-skyrmion scattering

This variety of dynamics of different skyrmionic spin structures in a temperature gradient is not captured by earlier works on this topic^30,31,43,44^. Specifically, the fact that the topologically trivial chimera skyrmion moves towards the cold regime, in connection with a finite transverse velocity, has not been captured so far. We will now demonstrate that all these peculiar features of the skyrmion Seebeck and the skyrmion Nernst effect as reported above can be explained by linear momentum transfer of an effective magnon current^34^ – due to the magnonic spin Seebeck effect^24,33^ – to the skyrmionic spin structures. Firstly, we show by means of spin model simulations that monochromatic magnon currents drive the skyrmionic spin structures in the same directions as a temperature gradient and, secondly, we employ scattering theory in connection with Thiele’s equation to identify linear momentum conservation as the relevant driving mechanism.

Magnon-skyrmion scattering can be studied in simulations by imposing forced oscillations of frequency $\omega$ and a fixed amplitude ${\tilde{A}}=\sqrt{S_x^2+S_y^2}\approx {2.5\times 10^{-2}}$ on the spins along the edge of the system. This produces magnons with wave vector $\mathbf {k}(\omega )$ that propagate perpendicular to this edge. Upon encountering the skyrmionic spin structures, the magnons are scattered and the skyrmion starts to move with a velocity $\mathbf {v}$. The direction anti-parallel to the wave vector $\mathbf {k}$ of the incident magnon and the skyrmion velocity $\mathbf {v}$ enclose an angle, which will be referred to as *skyrmion Nernst angle* $\theta$. Figure 2a depicts the skyrmion Nernst angle for the skyrmion, the antiskyrmions and the chimera skyrmion as a function of the absolute value of the wave vector $\mathbf {k}$ of the monochromatic magnon current. The results is compared to the skyrmion Nernst angle obtained for the temperature gradient motion at different values of $|\mathbf {\nabla } T|$. Note, that since the thermal magnon current induced by a temperature gradient travels from the hot to the cold end^33^, the skyrmion Nernst angle in a temperature gradient has to be defined as the angle between the skyrmion velocity and $\mathbf {\nabla }T$, in order to ensure consistency. Open symbols in Fig. 2a correspond to the values obtained from the simulations and the dotted lines are the semi-analytic predictions based on scattering theory that will be discussed later on.

Figure 2a shows that the values for the skyrmion Nernst angles of magnon and temperature gradient driven motion are in the same range, indicating that they are of common origin. The magnon current induced by a temperature gradient is a superposition of monochromatic magnon currents which are weighed by their respective occupation numbers (classically $\hbar \omega /k_{\mathrm {B}}T$) and group velocities. For finite damping, one also needs to take into account the finite propagation lengths of the magnon modes. This was done for example in a recent study of ferrimagnetic domain wall motion in a temperature gradient^45^. Albeit we do not follow this procedure here, we are nonetheless convinced that the results obtained for the magnon driven motion at hand provide sufficient evidence that magnon scattering dictates the directions of the motion of skyrmionic spin structures in a temperature gradient as well, because these are in qualitative agreement regarding the motion towards either the hot or the cold region or the skyrmion Nernst angle.

We will now proceed by demonstrating that linear momentum conservation is the relevant mechanism for the skyrmion Seebeck and Nernst effect. For that purpose, we employ spin wave scattering theory together with a rigid body approach for the skyrmion dynamics based on Thiele’s equation^38^, $G\mathbf {e}_\perp \times \mathbf {v}+\alpha D\mathbf {v}=\mathbf {F}$, with the gyrocoupling $G=-4\pi \mu _{\mathrm {s}}Q/(a^2\gamma )$ and the trace of the dissipation tensor $D=\mu _{\mathrm {s}}/(2a^2\gamma )\int (\partial _x \mathbf {S})^2+(\partial _y \mathbf {S})^2\mathrm {d}^2r$ and $\mu _{\mathrm {s}}$, $\gamma$ and *a* being the magnetic spin moment, the absolute value of the gyromagnetic ratio and the lattice constant. Due to the complexity of the skyrmionic spin structures, an incoming magnon current is not only deflected in a single direction. The fraction of the magnon current that is scattered in a certain direction is given by the differential cross section $\partial \sigma /\partial \chi$, $\chi$ being the scattering angle. By assuming elastic scattering and that the skyrmionic spin structure has a much larger mass than the magnon, the effective momentum transfer force resulting from a monochromatic magnon current with $\mathbf {k}=(k,0)^\mathrm {T}$ is given by (see Supplemental Material for the derivation)

1 $$\begin{aligned} \mathbf {F} = |A|^2 \hbar k \frac{\partial \omega }{\partial k} \int _0^{2\pi } \mathrm {d}\chi \ \bigg [ \begin{pmatrix} 1-\cos \chi \\ -\sin \chi \end{pmatrix} \dfrac{\partial \sigma }{\partial \chi } \bigg ]= |A|^2 \hbar k \frac{\partial \omega }{\partial k} \begin{pmatrix} \sigma _\parallel \\ \sigma _\perp \end{pmatrix} \end{aligned}$$

where *A* is the amplitude of the spin wave, $\sigma _\parallel /\sigma _\perp$ is the longitudinal/perpendicular cross section and $\partial \omega /\partial k$ is the group velocity. For a quadratic dispersion relation, $\omega \sim k^2$, the above formula (1) coincides with what was derived via a Lagrangian approach in an earlier study^46^. The effective momentum transfer force is determined by angular integrals over the differential cross section, weighted by $1-\cos \chi$ and $\sin \chi$. This indicates that no momentum is transferred for forward scattering ($\chi =0$) of the magnon. Furthermore, the component of $\mathbf {F}$ perpendicular to the wave vector is only finite for *skew scattering*, i.e. if the differential cross section is asymmetric with respect to $\chi$. Supplementing Eq. (1) to the Thiele equation yields

2 $$\begin{aligned} G\mathbf {e}_\perp \times \mathbf {v} +\alpha D\mathbf {v} =|A|^2 \hbar k \frac{\partial \omega }{\partial k} \begin{pmatrix} \sigma _\parallel \\ \sigma _\perp \end{pmatrix} \end{aligned}$$

and the skyrmion Nernst angle can be calculated from this expression via

3 $$\begin{aligned} \theta = \arctan \bigg ( \frac{-G \sigma _\parallel + \alpha D\sigma _\perp }{\alpha D \sigma _\parallel + G \sigma _\perp } \bigg ) \overset{\alpha D\ll G}{\approx } - \arctan \bigg ( \frac{\sigma _\parallel }{\sigma _\perp } \bigg ) \end{aligned}$$

The dynamics described by Eqs. (1), (2) and (3) are summarized in Fig. 2b: an incoming magnon is scattered at the core of the skyrmionic spin structure and transfers the momentum $\mathbf {p}^\mathrm {in}-\mathbf {p}^\mathrm {out}$, giving rise to an effective force $\mathbf {F}^\mathrm {mag}$. For a structure with finite *Q*, the second term (dissipation) in the Thiele Eq. (2) is usually much smaller than the first term (gyrocoupling) and thus the force is approximately perpendicular to the velocity. Depending on the sign of the topological charge, the direction of the velocity is obtained by either a clockwise or counterclockwise rotation by $\pi /2$. For the chimera skyrmion the gyrocoupling is zero and the force is henceforth parallel to the velocity.

The differential cross sections obtained from the simulations (for details on how to obtain them, see Supplemental Material) are shown in Fig. 2c as a function of spatial coordinates *x*, *y* and in Fig. 2d as a function of the scattering angle $\chi$. Multiple peaks in the differential cross section can be observed for all types of skyrmionic spin structures. The occurrence of multiple peaks, or *rainbow scattering*, is in agreement with analytical predictions for the $Q=1$ skyrmion^46^. We furthermore find a pronounced asymmetry with respect to forward scattering for skyrmionic spin structures with finite *Q*, giving rise to the so-called *topological magnon Hall effect*^47^, where the magnons are predominantly deflected to negative $\chi$ for skyrmions and to positive $\chi$ for antiskyrmions. For the chimera skyrmion we find three peaks: the forward scattering peak and one peak each for positive and negative $\chi$, respectively. We ascribe this to the fact that the chimera skyrmion consists of one region with positive topological charge and one region with negative topological charge: from a comparison with the differential cross sections obtained for skyrmions and antiskyrmions, we conclude that these regions lead to the peaks at negative and positive $\chi$. The asymmetry of the peaks in the differential cross section is a result of the symmetry-breaking between regions with positive and negative topological charges due to the DMI.

The resulting skyrmion dynamics can be understood from the differential cross sections using Eqs. (1) and (2). For the antiskyrmions the situation is as sketched in Fig. 2b. The scattering of the magnons to positive $\chi$ results in a force with negative transverse component. For small alpha it is $\mathbf {v}^\mathrm {sk/ask}\perp \mathbf {F}^\mathrm {mag}$ and, taking into account the sign of the topological charge, one obtains the direction of the force by rotation by $\pi /2$ in clockwise direction, giving rise to a longitudinal velocity that is opposite to the direction of the magnon current and a positive skyrmion Nernst angle. For the skyrmion, the situation is the opposite. Magnons are mainly scattered to negative $\chi$, the resulting force thus has a positive transverse component and the velocity is obtained via counter-clockwise rotation by $\pi /2$. This again results in a longitudinal motion anti-parallel to the direction of the incident magnon, but with a negative skyrmion Nernst angle. The situation for the chimera skyrmion is a little different. Since it has zero topological charge, the gyrocoupling term in Thiele’s equation vanishes and we get $\mathbf {v}^\mathrm {chim} = \mathbf {F}/(\alpha {\mathfrak {D}})$, so the velocity is in the same direction as the force. The two peaks of the differential cross section at positive and negative $\chi$ both lead to positive longitudinal forces, but the transversal forces have opposite sign. Since there is a slight asymmetry in the peaks, the individual contributions do not completely cancel each other and the transversal force attains a finite value. This explains why the chimera skyrmion has a small transverse velocity and, equivalently, why its skyrmion Nernst angle is not exactly $\pm \pi$

Finally, we come back to Fig. 2a. By virtue of Eq. (3), the skyrmion Nernst angle can be predicted solely from the differential cross sections (dotted lines). These predictions agree well with the simulation results (open symbols) and, hence, we identify linear momentum transfer of the magnon current to the skyrmionic spin structures—the basic assumption in the derivation of Eq. (1)—as the dominant contribution to the magnon driven skyrmion motion. These findings also imply that magnon scattering is the main contribution to the dynamics in a temperature gradient and, consequently, we conclude that the skyrmion Nernst angle depends on two aspects: the position of the scattering peaks of the thermal magnon current, i.e. the topological magnon Hall effect induced by the magnonic spin Seebeck effect, and the topological charge of the skyrmionic spin structure.

## Conclusion

In this article, we examined the dynamics of skyrmionic spin structures with a variety of topological charges driven either by a temperature gradient or monochromatic magnon currents by means of spin model calculations based on the stochastic Landau-Lifshitz-Gilbert equation. In a temperature gradient we found skyrmionic spin structures moving in different directions, depending on their topological charge: while skyrmions with finite $Q$ move towards the hot region, the topologically trivial chimera skyrmion drifts towards the cold region. Furthermore, the velocity perpendicular to the direction of the temperature gradient is nonzero for all types of skyrmions and its sign depends on the topological charge. We summarize the longitudinal and transverse dynamics under the terms skyrmion Seebeck effect and skyrmion Nernst effect, respectively.

Our findings are well explained by simulations of magnon-skyrmion scattering and a semi-analytic approach based on Thiele’s equation and spin wave scattering. We find that a monochromatic magnon current gives quantitatively similar results for the skyrmion Nernst angle as a temperature gradient and we identify linear momentum transfer as the dominant mechanism.

Our results reveal two pathways to experimentally determine the topological properties of localized spin structures. A temperature gradient could be used to sort them via their topological properties since localized structures with vanishing, positive and negative topological charge move in completely different directions. Moreover, the topology dependence of the skew scattering gives rise to the topological magnon Hall effect, which could be exploited to determine the topological properties via measuring the transverse component of a magnon current.

## Methods

### Atomistic spin model

We model a (Pt$_{0.95}$Ir$_{0.05}$)/Fe bilayer on a Pd(111) surface considering only the magnetic Fe moments. Our atomistic spin model is based on a spin Hamiltonian,

4 $$\begin{aligned} {\mathscr {H}} = \dfrac{1}{2} \sum _{i \ne j} \mathbf {S}_i{\mathscr {J}}_{ij}\mathbf {S}_j + \sum _i \mathbf {S}_i{\mathscr {K}} \mathbf {S}_i- \sum _i \mu _{\mathrm {s}}\mathbf {S}_i\cdot \mathbf {B}, \end{aligned}$$

for normalized magnetic moments $\mathbf {S}_i$. Ab initio values^48,49^ were used for the tensorial exchange coefficients ${\mathscr {J}}_{ij}$ (which model Heisenberg-exchange, Dzyaloshinsky-Moriya interaction and two-site anisotropy), the on-site anisotropy tensor ${\mathscr {K}}$ and the magnetic spin moment $\mu _{\mathrm {s}}$. The external magnetic field $\mathbf {B}$ is applied perpendicularly to the surface and has a fixed value of $B_{\perp } = 0.5 \mathrm{T}$ throughout the study. The ground state of the system is a spin spiral state which, upon application of a field of $B_{\perp } \geqslant 0.21\mathrm{T}$, transforms into a collinear state^20^. Skyrmionic spin structures with various topological charges $Q$ can then occur as metastable excitations. So far, skyrmionic spin structures with topological charges ranging from $-3$ to 3 have been identified^21^.

The time evolution of the spins is calculated by the means of the stochastic Landau-Lifshitz-Gilbert equation of motion^35^,

5 $$\begin{aligned} \dfrac{\partial {\mathbf {S}_i}}{\partial {t}} = - \dfrac{\gamma }{(1+\alpha ^2)\mu _{\mathrm {s}}}\mathbf {S}_i\times (\mathbf {H}_i+ \alpha \mathbf {S}_i\times \mathbf {H}_i) \end{aligned}$$

where $\alpha$ is the Gilbert damping parameter and $\gamma$ is the absolute value of the gyromagnetic ratio. The effective field $\mathbf {H}_i= -\partial {\mathscr {H}}/\partial \mathbf {S}_i+ \mathbf {\zeta }_i$ contains both the deterministic field resulting from the spin Hamiltonian (4) and the stochastic field $\mathbf {\zeta }_i$ which is characterized by $\langle \mathbf {\zeta }_i(t)\rangle = \mathbf {0}$ and $\langle \mathbf {\zeta }_i(t) \mathbf {\zeta }^{\mathrm {T}}_j(t')\rangle = 2 \alpha \mu _{\mathrm {s}}k_{\mathrm {B}}T_i \mathbbm{1}\delta _{ij} \delta (t-t')/\gamma$ with $k_{\mathrm {B}}$ being the Boltzmann constant. Note, that – since we are interested in temperature gradients – the temperature is position dependent, which is indicated by the index *i*.

The numerical integration of the stochastic Landau-Lifshitz-Gilbert equation is performed via a GPU-based implementation of the Heun algorithm^35^. We simulate with a fixed timestep $\Delta t = {{1.4\times 10^{-15}}s}$ and open boundary conditions. Unless stated otherwise, the temperature gradient is assumed to be in *x*-direction and has a fixed value of $\partial T/ \partial x= { 0.01}{\mathrm{K}}/\mathrm{a}$, with $a={2.751\times 10^{-10}}{\mathrm{m}}$ being the lattice constant, such that the local temperature is given by $T_i =(\partial T/ \partial x) x_i$. Since the system we simulate consists of ${{256}} \times {{128}}$ unit cells, the temperature difference between the hot and cold end of the system is then approximately ${3}{\mathrm{K}}$. As thermal skyrmion dynamics are stochastic, we perform multiple simulations (between eight and 50) for a given set of parameters and average over the resulting trajectories.

To study the magnon driven motion of skyrmionic spin structures, we simulate at zero temperature and excite monochromatic magnons via forced oscillations of frequency $\omega$ with fixed amplitude ${\tilde{A}}=\sqrt{S_x^2+S_y^2}\approx {2.5\times 10^{-2}}$ along one edge of the system, i.e. $S_z^\mathrm {edge}=\sqrt{1-{\tilde{A}}^2}$, $S_x^\mathrm {edge}= {\tilde{A}} \cos \omega t$ and $S_y^\mathrm {edge}= {\tilde{A}} \sin \omega t$.

## Supplementary Information

Supplementary Information.
