# Supplementary material for: Topology dependence of skyrmion Seebeck and skyrmion Nernst effect
Source: Sci Rep. 2022 Apr 26;12:6801. doi: 10.1038/s41598-022-10550-z (PMC9042842; doi:10.1038/s41598-022-10550-z)
Supplement: Supplementary file 1 — Supplementary Information. [file 41598_2022_10550_MOESM1_ESM.pdf]

Supplemental Material to ’Topology dependence of Skyrmion Seebeck and Skyrmion Nernst effect’

Scattering theory

In the following we discuss the scattering of a monochromatic magnon current at a localized spin structure and we derive, by using momentum conservation, the effective momentum transfer force exerted on the localized spin structure in the limit of vanishing damping. Without loss of generality, it is assumed that the spin structure is located at $\boldsymbol{r} = 0$ and that the magnetization far away from the localized spin structure is in z-direction.

Using the transformation $S_{\pm}(\boldsymbol{r},t) = S_x(\boldsymbol{r},t) \pm iS_y(\boldsymbol{r},t)$ the incoming (clock-wise) spin wave far away from the core of the localized spin structure can be written as

$$S_+^{\mathrm{in}}(\boldsymbol{r},t) = Ae^{i(\boldsymbol{k}\cdot\boldsymbol{r}-\omega t)}, \tag{1}$$

where A is the spin wave amplitude and the frequency ω is related to the wave vector $\boldsymbol{k}$ via the dispersion relation. Assuming that no energy is transferred (i.e. that the localized spin structure is much heavier than the magnon) the outgoing scattering wave function far away from the core can be written as[1]

$$S_+^{\mathrm{out}}(\boldsymbol{r},t) = Ae^{-i\omega t}\left(e^{i\boldsymbol{k}\cdot\boldsymbol{r}} + f(\chi)\frac{e^{ik\rho}}{\sqrt{\rho}}\right), \tag{2}$$

with $f(\chi)$ being the scattering amplitude and ρ, χ being polar coordinates. The total momentum of the incoming and outgoing spin waves can be obtained by using the momentum operator $\hat{\boldsymbol{p}} = (\hbar/i)\boldsymbol{\nabla}$ and read

$$\boldsymbol{p}^{\mathrm{in}} = \langle S_+^{\mathrm{in}}|\hat{\boldsymbol{p}}|S_+^{\mathrm{in}}\rangle = \int \left[(S_+^{\mathrm{in}})^*\frac{\hbar}{i}\boldsymbol{\nabla} S_+^{\mathrm{in}}\right]\mathrm{d}^2r = |A|^2\int \hbar\boldsymbol{k}\,\mathrm{d}^2r \tag{3}$$

$$\boldsymbol{p}^{\mathrm{out}} = \langle S_+^{\mathrm{out}}|\hat{\boldsymbol{p}}|S_+^{\mathrm{out}}\rangle = \int \left[(S_+^{\mathrm{out}})^*\frac{\hbar}{i}\boldsymbol{\nabla} S_+^{\mathrm{out}}\right]\mathrm{d}^2r \approx |A|^2\int \left[\hbar\boldsymbol{k} + \frac{\hbar k|f(\chi)|^2}{\rho}\boldsymbol{e}_\rho - \frac{\hbar|f(\chi)|^2}{\rho}\boldsymbol{k} + \mathscr{O}(\rho^{-3/2})\right]\mathrm{d}^2r \tag{4}$$

with $\boldsymbol{e}_\rho = (\cos\chi, \sin\chi)^{\mathrm{T}}$ and where rapidly fluctuating contributions proportional to $\exp(\pm i\boldsymbol{k}\cdot\boldsymbol{r})\exp(\mp ik\rho)$ are neglected as they average to zero. Note that the two-dimensional optical theorem[2] was used in the derivation of the above expressions. By the virtue of momentum conservation, the total momentum transferred to the localized spin structure is hence given by

$$\Delta\boldsymbol{p} = \boldsymbol{p}^{\mathrm{in}} - \boldsymbol{p}^{\mathrm{out}} = |A|^2\hbar k\int_0^\infty \mathrm{d}\rho \int_0^{2\pi}\mathrm{d}\chi \left[\begin{pmatrix}1-\cos\chi\\ -\sin\chi\end{pmatrix}\frac{\partial\sigma}{\partial\chi} + \mathscr{O}(\rho^{-1/2})\right] \tag{5}$$

where it was used that $|f(\chi)|^2 = \partial\sigma/\partial\chi$ and the wave vector of the incoming wave was assumed to be in x-direction, i.e. $\boldsymbol{k} = (k,0)^{\mathrm{T}}$. In order to calculate the effective force that stems from this momentum transfer, one needs to evaluate the flow of momentum through a circle around the core of the localized spin structure. Since the flow of momentum through such a circle does not depend on the radius (if damping is neglected), it is convenient to choose a large radius because then the terms with $\mathscr{O}(\rho^{-1/2})$ in the above expression become negligible. Within the time interval Δt, only the part of the spin wave that is at most $\Delta t v$ (with $v = \partial\omega/\partial k$ being the group velocity) away from this circle contributes to the flow of momentum. So the flow of momentum through a large circle with radius R around the core within the time interval Δt is given by

$$\Delta\boldsymbol{p}(\Delta t)|_R \approx |A|^2\hbar k\int_{R-v\Delta t}^{R}\mathrm{d}\rho \int_0^{2\pi}\mathrm{d}\chi \left[\begin{pmatrix}1-\cos\chi\\ -\sin\chi\end{pmatrix}\frac{\partial\sigma}{\partial\chi}\right] = |A|^2\hbar k v\Delta t\int_0^{2\pi}\mathrm{d}\chi \left[\begin{pmatrix}1-\cos\chi\\ -\sin\chi\end{pmatrix}\frac{\partial\sigma}{\partial\chi}\right]. \tag{6}$$

Finally, the effective momentum transfer force exerted on the localized spin structure follows as

$$\boldsymbol{F} = \lim_{\Delta t\to 0}\frac{\Delta\boldsymbol{p}(\Delta t)|_R}{\Delta t} = |A|^2\hbar k\frac{\partial\omega}{\partial k}\int_0^{2\pi}\mathrm{d}\chi \left[\begin{pmatrix}1-\cos\chi\\ -\sin\chi\end{pmatrix}\frac{\partial\sigma}{\partial\chi}\right] = |A|^2\hbar k\frac{\partial\omega}{\partial k}\begin{pmatrix}\sigma_\parallel\\ \sigma_\perp\end{pmatrix} \tag{7}$$

with $\sigma_\parallel$ being the longitudinal cross section and $\sigma_\perp$ being the perpendicular cross section. If a quadratic dispersion relation is assumed, i.e. $\omega \sim k^2$, the above formula (7) coincides with what was derived via a Lagrangian approach in an earlier study[1].

The differential cross section $\partial\sigma/\partial\chi$ can be expressed in terms of spin components by using equations (1) and (2) and the fact that $S_+^{\mathrm{in/out}}(\rho,\chi) = S_x^{\mathrm{in/out}}(\rho,\chi) + iS_y^{\mathrm{in/out}}(\rho,\chi)$, yielding

$$\frac{\partial\sigma}{\partial\chi}(\chi) = |f(\chi)|^2 = \rho\,\frac{(S_x^{\mathrm{out}}(\rho,\chi) - S_x^{\mathrm{in}}(\rho,\chi))^2 + (S_y^{\mathrm{out}}(\rho,\chi) - S_y^{\mathrm{in}}(\rho,\chi))^2}{(S_x^{\mathrm{in}}(\rho,\chi))^2 + (S_y^{\mathrm{in}}(\rho,\chi))^2}. \tag{8}$$

Following equation (7), the *direction* of the effective momentum transfer force can henceforth be calculated from the spin components at a given time. We exploit this fact in our investigation of the scattering of a monochromatic magnon current at a skyrmionic spin structure: when simulating magnon-skyrmion scattering (from which we obtain the spin components of the scattered wave function), we perform a duplicate simulation in the absence of the skyrmionic spin structure (from which we obtain the spin components of the unscattered wave function). This allows us to calculate the value of the differential cross section at each lattice site. By selecting all values of $\partial\sigma/\partial\chi$ within an annulus around the skyrmionic spin structure we obtained the differential cross section as a function of the scattering angle χ.

References

1. Schütte, C. & Garst, M. Magnon-skyrmion scattering in chiral magnets. *Phys. Rev. B* **90**, 094423, DOI: 10.1103/PhysRevB.90.094423 (2014).

2. Panja, M. M., Bera, P. K. & Talukdar, B. Optical theorem and aharonov-bohm scattering. *Pramana* **45**, 499–509, DOI: 10.1007/BF02848173 (1995).
